# Genetic Variability of Inflammation and Oxidative Stress Genes Affects Onset, Progression of the Disease and Survival of Patients with Amyotrophic Lateral Sclerosis

**DOI:** 10.3390/genes13050757

**Published:** 2022-04-25

**Authors:** Metka Ravnik-Glavač, Katja Goričar, David Vogrinc, Blaž Koritnik, Jakob Gašper Lavrenčič, Damjan Glavač, Vita Dolžan

**Affiliations:** 1 Institute of Biochemistry and Molecular Genetics, Faculty of Medicine, University of Ljubljana, 1000 Ljubljana, Slovenia; katja.goricar@mf.uni-lj.si (K.G.); david.vogrinc@mf.uni-lj.si (D.V.); vita.dolzan@mf.uni-lj.si (V.D.); 2 Institute of Clinical Neurophysiology, Division of Neurology, University Medical Centre Ljubljana, 1000 Ljubljana, Slovenia; blaz.koritnik@kclj.si; 3 Department of Neurology, Faculty of Medicine, University of Ljubljana, 1000 Ljubljana, Slovenia; 4 Department of Molecular Genetics, Institute of Pathology, Faculty of Medicine, University of Ljubljana, 1000 Ljubljana, Slovenia; jakob.lavrencic@gmail.com (J.G.L.); damjan.glavac@mf.uni-lj.si (D.G.); 5 Center for Human Genetics & Pharmacogenomics, Faculty of Medicine, University of Maribor, 2000 Maribor, Slovenia

**Keywords:** amyotrophic lateral sclerosis, ALS, single-nucleotide polymorphisms, genotyping, inflammation, oxidative stress, clinical modifiers

## Abstract

Inflammation and oxidative stress are recognized as important contributors to amyotrophic lateral sclerosis (ALS) disease pathogenesis. Our aim was to evaluate the impact of selected single-nucleotide polymorphisms in genes involved in inflammation and oxidative stress on ALS susceptibility and modification. One-hundred-and-eighty-five ALS patients and 324 healthy controls were genotyped for nine polymorphisms in seven antioxidant and inflammatory genes using competitive allele-specific PCR. Logistic regression; nonparametric tests and survival analysis were used in the statistical analysis. Investigated polymorphisms were not associated with ALS susceptibility. Carriers of at least one polymorphic *SOD2* rs4880 T or *IL1B* rs1071676 C allele more often had bulbar ALS onset (*p* = 0.036 and *p* = 0.039; respectively). *IL1B* rs1071676 was also associated with a higher rate of disease progression (*p* = 0.015). After adjustment for clinical parameters; carriers of two polymorphic *IL1B* rs1071676 C alleles had shorter survival (HR = 5.02; 95% CI = 1.92–13.16; *p* = 0.001); while carriers of at least one polymorphic *CAT* rs1001179 T allele had longer survival (HR = 0.68; 95% CI = 0.47–0.99; *p* = 0.046). Our data suggest that common genetic variants in the antioxidant and inflammatory pathways may modify ALS disease. Such genetic information could support the identification of patients that may be responsive to the immune or antioxidant system—based therapies.

## 1. Introduction

Amyotrophic lateral sclerosis (ALS) is characterized by progressive degeneration of both upper and lower motor neurons, resulting in muscle weakness, atrophy, and gradual paralysis, leading to death due to respiratory failure usually within 2–3 years following the onset of symptoms [1]. The disease most typically begins in the limbs (spinal onset), but in approximately 20% of cases, oropharyngeal muscles weakness (bulbar onset) is the first evident symptom of the disease [2]. ALS has an incidence of 2–3 per 100,000 and a lifetime risk of 1 per 400 individuals [3], with some differences between populations [4].

About 5–10% of ALS cases have a familial background (FALS), but the vast majority are categorized as sporadic ALS (SALS) based on negative family history. FALS and SALS are mostly phenotypically indistinguishable, suggesting common pathways exist at the basis of neuronal death. In SALS, the lack of positive family history for ALS could be due to incomplete penetrance or oligogenic/polygenic inheritance [5].

To date, several genetic factors have been identified that drive motor neuron degeneration in ALS, increase susceptibility to the disease, or influence the rate of progression [6,7]. In the ALS database, around 150 genes have been reported that are, according to their contribution to ALS pathogenesis, classified as definitive ALS genes, clinical modifiers, and genes with strong or moderate evidence (https://alsod.ac.uk/. accessed on 15 March 2022). Although more than 30 of these genes strongly correlate with the disease, their exact roles in disease pathogenesis are still not completely understood [8]. The main proposed interrupted mechanisms by most genes mutated in ALS include disrupted proteostasis by increased protein aggregation (*SOD1*, *TARDBP*, *FUS*, *C9orf72*) decreased proteasomal degradation (*VCP*, *UBQLN2*), or impaired autophagy (*OPTN*, *TBK1*, *CYLD*, *C9orf72*, *ALS2*, *SQSTM1*, *VCP*, *UBQLN2*, *CHMP2B*). Several of these genes have also multiple functions. For example, mutations in *SOD1* and *SQSTM1* can lead to mitochondrial damage and oxidative stress [8].

Oxidative stress is a process where an accumulation of reactive oxygen species (ROS) leads to cellular damage and cell death due to an imbalance between free radical production and antioxidant defenses [9,10,11]. Cellular ROS levels may be reduced through the defense mechanisms of antioxidant enzymes [12]. Of these, manganese SOD (*SOD2*) is mainly involved in the elimination of highly reactive O_2_^−^ in the cytosol and mitochondria to produce H_2_O_2_ [13]. Then H_2_O_2_ may be further removed by the action of glutathione peroxidases (GPX) and catalase (CAT) [14]. Studies have suggested that upregulation of GPX1 could be one of the protective responses against neuronal injury [14]. CAT is located mainly in peroxisomes and is responsible for the conversion of H_2_O_2_ to water and oxygen [12,13]. The contribution of catalase to the oxidative stress response is minor at low levels of H_2_O_2_ but becomes increasingly important at higher levels of H_2_O_2_ [12,15].

Oxidative stress is thought to increase with age, a major risk factor in ALS [16]. High levels of ROS can damage several different parts of the cellular machinery through lipid peroxidation and oxidation of proteins and/or DNA. In SOD1 G93A rodent ALS models as well as cell ALS models, researchers found oxidative damage to DNA, RNA, proteins, and lipids [17]. Markers of ROS damage have also been reported to be increased in cerebrospinal fluid, plasma, serum, and urine of SALS patients [18,19,20,21] and in post-mortem ALS spinal cord tissue [11,22,23,24].

Excessive production of ROS can result in reduced efficiency of cellular processes, excitotoxicity, protein aggregation and endoplasmic reticulum stress, and induction of inflammatory pathways that have all been directly involved in disease pathogenesis [9,15]. Inflammation/neuroinflammation has also been recognized as an important mediator of the pathogenesis of disease in ALS [25]. Inflammation can be triggered by aggregated proteins and impaired autophagy and/or oxidative stress and mitochondrial damage. The link between inflammation and other proposed ALS-related mechanisms is thus complex and multidirectional and can at some point initiate a vicious cycle of a pathogenic cascade of molecular events that gradually damages motor neurons to the point that cell deterioration is irreversible [8,26]. In addition, mutations in several genes that have been reported in ALS patients, including *TBK1*, *OPTN*, *CYLD,* and *C9orf72*, are directly linked to the immune response [8,27,28,29,30].

Several studies indicated that deregulation of immune response may in many cases occur early in the course of ALS. Namely, in mouse ALS models longitudinal live imaging studies revealed, in the very early presymptomatic stages of the disease, significant changes in activation of astrocytes and microglia [31,32]. Microglia are a central protagonist of the neuroinflammatory component of neurodegeneration in ALS. Macrophages from activated microglia are largely pro-inflammatory and secrete a number of proinflammatory cytokines such as tumor necrosis factor α (TNF-α), interferon γ (IFNγ), and interleukin 1β (IL-1β) [25]. In animal models, specific deletion of C9orf72 from myeloid cells in mice (specifically macrophages and microglia) resulted in lysosomal accumulation, hyperactive immune responses, and increased expression of cytokines IL-6 and IL-1β, altering the normal immune function of these cells [33].

Increasing evidence suggests that inflammatory response in the central nervous system (CNS) that includes proinflammatory cytokines contributes to the pathogenesis of ALS also in human patients [34]. Positron emission tomography imaging studies together with studies of proteins expressed by activated microglia revealed active gliosis in patients with ALS in vivo and demonstrated widespread microglial activation in the motor cortex, dorsolateral prefrontal cortex, and thalamus [35,36,37]. Increased microgliosis correlated positively with Upper Motor Neuron scores and negatively with ALS Functional Rating Scores (ALS-FRS) [35,38] thus indicating a positive relationship between neuroinflammation and disease severity. Infiltration of immune cells was observed in the CNS of ALS patients at sites of motor neuron injury, including macrophages and T-cells [39,40,41]. In addition, peripheral immune abnormalities including T-cells, cytokines, chemokines, and other markers of inflammation have been detected in blood in clinical studies [42]. ALS monocytes demonstrated a unique inflammation-related gene expression profile, including increased *IL1B* and *IL8* expression [43].

Recent work further suggests that microglial release of inflammatory factors serves as a trigger for activated neurotoxic astrocytes [44]. Additionally, some miRNAs have been found to be involved in this process. For example, miR-146a was upregulated in IL-1β-stimulated human astrocytes and was associated with the regulation of an astrocyte-mediated inflammatory response [45]. Downregulation of miR-146a affected TLR/NF-κB signaling pathways in murine SOD1 astrocytes, thus contributing to neuroinflammation [46]. Banack et al. recently reported the upregulation of miR-146a-5p in the neural enriched extracellular vesicles of ALS patient samples compared to healthy controls [47].

The potential causative or disease driving role of inflammation and oxidative stress in ALS is not fully understood, as there are studies that support both a primary and secondary role during ALS disease progression [9,48]. Genetic factors can affect both inflammation and response to oxidative stress, but the role of common polymorphisms in ALS susceptibility or pathogenesis has not been well established. In this study, we aimed to further elucidate the role of inflammation and oxidative stress in the pathogenesis of ALS by genotyping ALS patients and controls for common genetic polymorphisms in genes involved in antioxidant (*SOD2*, *GPX*, and *CAT*) and inflammatory pathways (*IL1B*, *TNF*, *IL6*, *MIR146A*).

## 2. Subjects and Methods

### 2.1. Subjects

The patient cohort consisted of 185 SALS patients diagnosed and collected between 2012 and 2019 at the tertiary Ljubljana ALS Centre which takes care of the majority of Slovenian ALS patients. Patients’ age was 56 to 71 years and there were 94 male and 91 female (Table 1). In our previous study, we screened 85 of these SALS patients for common mutations in four major ALS-associated genes, *SOD1*, *TARDBP*, *FUS*, and *C9orf72* [49]. Functional impairment of the patients was assessed routinely by the ALS-FRS-R during the out-patient visits every three months. ALS-FRS-R data were not available for 21 patients who did not have an out-patient visit at the time of blood collection. We obtained approval for this study from the National Medical Ethics Committee of the Republic of Slovenia (Approval number: 68/12/13 and 0120-120/2018/8) and all participants provided written informed consent. The Control group consisted of 324 unrelated healthy Slovenian blood donors without any systemic disease aged 40 to 65 years, 238 were male, and 86 were female.

### 2.2. DNA Extraction and Genotyping

DNA from patients’ peripheral venous blood samples was isolated using QIAamp Blood Midi Kit (Qiagen, Hilden, Germany) following the manufacturer’s protocol. For healthy controls, genomic DNA was isolated from peripheral venous blood samples using Qiagen FlexiGene Kit (Qiagen, Hilden, Germany) according to the manufacturer’s instructions.

Genotyping was performed for nine single nucleotide polymorphisms (SNPs) with known or predicted functional effects across seven genes important in antioxidant and inflammatory pathways [50]. *SOD2* (rs4880), *CAT* (rs1001179), *GPX1* (rs1050450), *IL1B* (rs1143623, rs16944, rs1071676), *MIR146A* (rs2910164), *IL6* (rs1800795) and *TNF* (rs1800629) were genotyped using a fluorescent-based, competitive allele-specific polymerase chain reaction (KASP, LGC Genomics, Hoddesdon, UK) according to the manufacturer’s protocol. For quality control, 10% of samples were genotyped in duplicate and all results were concordant.

Genotype frequencies of all nine investigated SNPs in *SOD2*, *CAT*, *GPX1*, *IL1B*, *IL6*, and *TNF* genes among ALS patients and healthy controls are presented in Supplementary Table S1. Genotype frequencies of all investigated SNPs were in agreement with Hardy-Weinberg Equilibrium in both controls and ALS patients.

### 2.3. Statistical Analysis

Continuous variables were described with median and interquartile range (25–75%), while categorical variables were described with frequencies. Deviation from the Hardy-Weinberg equilibrium (HWE) was evaluated using the chi-square test. Both dominant and additive genetic models were used in the analysis. Logistic regression was used to evaluate the association of selected SNPs with binary categorical variables and to determine the odds ratios (ORs) and their 95% confidence intervals (CIs). Fisher’s exact test was used if there were no subjects within one of the groups or for dependent categorical variables with more than two categories, as well as to compare different subject groups. Nonparametric Mann-Whitney or Kruskal-Wallis test with *post hoc* Bonferroni corrections for pairwise comparisons were used to evaluate the association of SNPs with continuous variables.

Survival of ALS patients was defined as the time from disease onset to death. Patients without death at the time of the analysis were censored at the date of the last follow-up. Kaplan-Meier test was used to calculate median survival or follow-up time, while Cox regression was used to assess the role of selected SNPs and to calculate the hazard ratios (HR) with their 95% CIs. Clinical parameters used for adjustment in multivariable regression models were selected using stepwise forward-conditional regression. IBM SPSS Statistics version 27.0 (IBM Corporation, Armonk, NY, USA) was used for all analyses. All tests were two-sided, and the level of significance was set at 0.05.

## 3. Results

We included in our study 185 ALS patients and 324 healthy controls. The level of functional impairment according to ALS-FRS-R data was available for 164 ALS patients. Clinical characteristics of all ALS patients and patients with available ALS-FRS-R data are presented in Table 1. Most patients had spinal onset ALS (133, 72.3%) and 153 (86.0%) died by the time of the analysis. *C9orf72* mutation was found in six (3.2%) patients. Median survival was 39.7 (26.6–68.4) months and median follow-up was 104.9 (90.7–156.6) months.

Among healthy controls, 238 (73.5%) were male and 86 (26.5%) female. The gender distribution differed significantly between healthy controls and ALS patients (*p* = 0.001). The median age of healthy controls was 49 (44–55) years, which was significantly younger compared to ALS patients (*p* < 0.001).

### 3.1. Association of Investigated SNPs with ALS Susceptibility and ALS Type

None of the investigated SNPs was associated with ALS susceptibility, not even after adjustment for gender and age (Table 2).

On the other hand, some of the genotype frequencies differed between groups of patients with spinal, bulbar, and other ALS types at disease onset (Table 3). Carriers of at least one polymorphic *SOD2* rs4880 T allele more often had bulbar onset ALS when compared to carriers of two C alleles that had more often spinal onset ALS (*p* = 0.036). Similarly, bulbar onset ALS was more frequent in carriers of at least one polymorphic *IL1B* rs1071676 C allele compared to carriers of two G alleles (*p* = 0.039). Conversely, spinal onset ALS was more frequent in carriers of at least one polymorphic *IL1B* rs16944 C allele, while other ALS types were more common in carriers of two T alleles, but the difference did not reach statistical significance (*p* = 0.051).

### 3.2. Association of Investigated SNPs with Level of Functional Impairment and Rate of Disease Progression in ALS Patients

**Figure 1:**
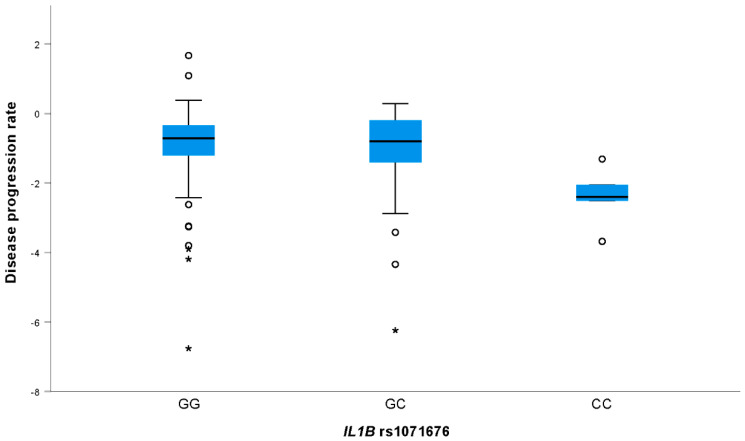
Association of *IL1B* rs1071676 with ALS-FRS-R points per month progression rate. In the boxplots, empty circles represent outliers between 1.5 and 3-times interquartile range away from the 1st or 3rd quartiles, while outliers beyond 3-times interquartile range are presented with stars (*).

Among the investigated SNPs, only *IL1B* rs1071676 was associated with the rate of disease progression (*p* = 0.015, Table 4, Figure 1). After adjustment for multiple comparisons, carriers of two polymorphic *IL1B* rs1071676 C alleles had a higher rate of disease progression compared to carriers of two G alleles (P_adj_ = 0.015) and heterozygotes (P_adj_ = 0.012).

However, none of the investigated SNPs was associated with a level of functional impairment in ALS patients in our study (Table 4).

### 3.3. Association of Investigated SNPs with Survival of ALS Patients

Among the investigated clinical parameters, shorter survival was observed in older patients (HR = 1.04, 95% CI = 1.02–1.06, *p* < 0.001). Bulbar onset ALS (HR = 1.85, 95% CI = 1.27–2.69, *p* = 0.001) and other onset ALS types (HR = 3.19, 95% CI = 1.38–7.35, *p* = 0.007) were associated with shorter survival compared to spinal onset ALS. Slower rate of disease progression was also significantly associated with longer survival (HR = 0.60, 95% CI = 0.53–0.68, *p* < 0.001). In forward conditional analysis, age at onset (HR = 1.04, 95% CI = 1.02–1.05, *p* < 0.001) and rate of disease progression (HR = 0.63, 95% CI = 0.56–0.72, *p* < 0.001) remained associated with survival and were used for adjustment in multivariable models.

**Figure 2:**
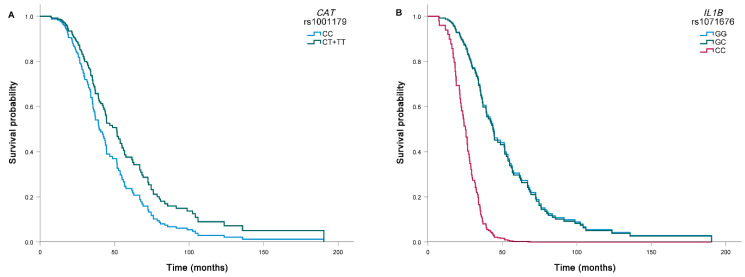
Association of *CAT* rs1001179 (**A**) and *IL1B* rs1071676 (**B**) with survival of ALS patients. Carriers of at least one polymorphic *CAT* rs1001179 T allele had longer survival after adjustment for age at onset and rate of disease progression (HR = 0.68, 95% CI = 0.47–0.99, *p* = 0.046). Carriers of two polymorphic *IL1B* rs1071676 C alleles had shorter survival after adjustment for age at onset and rate of disease progression (HR = 5.02, 95% CI = 1.92–13.16, *p* = 0.001).

In univariable analysis, none of the investigated SNPs was significantly associated with the survival of ALS patients (Table 5). Carriers of at least one polymorphic *CAT* rs1001179 T allele had longer survival compared to carriers of two C alleles (47.6 months compared to 37.0 months), but the association was only significant after adjustment for clinical parameters (HR = 0.68, 95% CI = 0.47–0.99, *p* = 0.046; Figure 2A). Carriers of two polymorphic *IL1B* rs1071676 C alleles had shorter survival compared to carriers of two G alleles (24.3 months compared to 39.7 months), but the association was only significant after adjustment for clinical parameters (HR = 5.02, 95% CI = 1.92–13.16, *p* = 0.001; Figure 2B).

## 4. Discussion

Inflammation/neuroinflammation and oxidative stress are important mediators of the pathogenesis of disease in ALS and have both been reported to be involved in the early stages of the disease [8,9]. However, results that indicate a causative or significant role of oxidative stress and immune system components in ALS pathogenesis are limited and more results support their involvement in exacerbating the disease progression [9,48,51]. Although oxidative stress and inflammation are probably not the main triggers of the disease, an important challenge to ALS research is also to find out how endogenous genetic variants of inflammation and antioxidant factors modify the disease that accounts for different disease courses. To further elucidate this challenging topic, we have genotyped a representative cohort of SALS patients and controls for common genetic polymorphisms in genes involved in antioxidant and inflammatory pathways. In the present study, *IL1B* rs1071676 polymorphism was associated with disease onset, rate of disease progression, and survival of ALS patients. Selected polymorphisms in antioxidant genes were also associated with some clinical characteristics, while no association with ALS susceptibility was observed. To our knowledge, these selected polymorphisms have not been previously studied in connection with ALS.

Our results revealed that none of the investigated SNPs was associated with ALS disease risk. Although there are few studies on how polymorphisms in oxidative stress response and inflammatory pathways influence ALS pathogenesis, they are all in agreement with our results. In previous studies investigating antioxidant genes, mutation, or individual common polymorphisms in *SOD2*, glutathione S-transferase P1 (*GSTP1*) or paraoxonase 1 (*PON1*) genes were not associated with ALS susceptibility [52,53,54]. Regarding inflammatory processes, previous studies did not investigate genetic variability in the genes included in our study. On the other hand, functional variants of the human *CX3CR1* gene, also known as fractalkine receptor, were also not associated with ALS disease risk [55,56]. Fractalkine signaling affects inflammatory responses and has been discovered to play a role in the migration of microglia to their synaptic targets, where phagocytosis and synaptic refinement occur during the development of the central nervous system [57].

The key result of our study is the association of *IL1B* rs1071676 with several different patients’ characteristics: disease onset, rate of disease progression, and survival of ALS patients. On the other hand, no significant associations with ALS were observed for common *IL1B* promotor polymorphisms rs1143623 and rs16944.

*IL1B* rs1071676 occurs in the 3′-UTR region (c.*505G > C) of the gene and is predicted to alter the miRNA binding site with potential functional effect [58]. In our study, carriers of two polymorphic *IL1B* rs1071676 C alleles had a higher rate of disease progression compared to carriers of two G alleles and heterozygotes. Similarly, carriers of two polymorphic *IL1B* rs1071676 C alleles had a 15.4-month shorter median survival compared to carriers of two G alleles, but the association was only significant after adjustment for clinical parameters. In addition, carriers of at least one polymorphic *IL1B* rs1071676 C allele had more bulbar onset of ALS. Consistently, patients with bulbar onset had shorter survival compared to patients with spinal ALS onset. To the best of our knowledge, this polymorphism was previously not investigated in ALS and its role is not well known. However, it was previously already associated with brain patterns after hypoxic-ischemic encephalopathy [59], fatigue in adults with HIV/AIDS [60], and response to anti-TNF therapy in Crohn’s disease [58]. Further studies are therefore needed to elucidate the role of this polymorphism in IL-1β expression.

On the other hand, the role of IL-1β in ALS was already previously reported. The NLRP3 inflammasome is required for the activation of caspase 1 and the processing and release of IL-1β and IL-18 [61]. Johann et al. investigated the expression of the NLRP3 inflammasome in SOD1 mutated ALS animal model and in post-mortem tissue of ALS patients [61]. They detected NLRP3 and IL-1β in spinal cord astrocytes already at a pre-symptomatic stage in SOD1 mutant mice. NLRP3 components and active caspase 1 levels were also increased in human ALS tissue compared to controls, which suggested that astroglial NLRP3 inflammasome complexes may contribute to neuroinflammation in ALS [61]. In SOD1 mutated mice models, an increase in IL-1β levels was consistently reported [62,63,64], further suggesting an association with disease progression through the promotion of neuroinflammation [62]. IL-1β was even proposed as a potential therapeutic target [62]. However, the results of studies evaluating blood or cerebrospinal fluid IL-1β in ALS patients were not always consistent [65,66,67]. Still, several studies observed an increase of IL-1β in ALS patients, even though its levels were not always above the limit of detection [65,66]. Importantly, increased IL-1β was observed in ALS patients with short survival, and increased expression was associated with shorter survival, especially in carriers of *C9orf72* mutation [65]. These results are consistent with our results, suggesting IL-1β variability should be further examined in larger studies.

Among polymorphisms in antioxidant genes, we observed in our cohort differences in *SOD2* rs4880 genotype frequencies distribution between spinal, bulbar, and other ALS onsets. Carriers of at least one polymorphic *SOD2* rs4880 T allele more often had bulbar onset of ALS compared to carriers of two C alleles that had more spinal onset of ALS. This single C/T nucleotide polymorphism is the most characterized polymorphism in the signal sequence of the *SOD2* gene that results in p.Ala16Val of the mitochondrial targeting sequence in the *SOD2* protein. It was predicted that the valine-containing protein (rs4880 T allele) would form a β-sheet rather than the expected α-helix of alanine-containing protein (rs4880 C allele). It was shown, consistent with this prediction, that the p.16Val *SOD2* had reduced import efficiency into the mitochondria as well as reduced mRNA stability and activity in the mitochondria, which in turn both increase oxidative stress [68,69,70]. It was also predicted that the association of *SOD2* rs4880 gene polymorphism with various different diseases may be due to differential modulation of oxidative stress with other endogenous as well as exogenous antioxidants and may thus be partly influenced by environmental factors, like physical activity and diet [68,70,71]. However, further studies are needed to better evaluate the role of *SOD2* polymorphisms in ALS. Verde et al. [72] reported similar results for rs662 (Q192R) in detoxifying enzyme *PON1*. They found *PON1* minor allele G to be associated both with the bulbar onset and independently with reduced survival. Q192R modifies enzyme activity with regard to substrate specificity and catalytic efficiency. Authors hypothesized that 192R alloenzyme is less efficient in ameliorating certain endogenous damaging processes such as oxidative stress or in metabolizing some exogenous toxic substances [72].

Catalase plays a significant role in protecting cells against severe oxidative stress [12]. In our study, carriers of at least one polymorphic *CAT* rs1001179 T allele had significantly longer survival compared to carriers of two C alleles after adjustment for clinical parameters. This common C/T polymorphism is located 262 base pairs upstream from the transcription start site of the catalase gene and affects transcription factor binding and alters gene expression [73,74]. In blood samples, catalase levels were significantly higher in donors carrying the T allele in comparison to donors homozygous for the C allele [73], in concordance with our study, where this allele was associated with longer survival. Studies investigating this polymorphism in neurodegenerative diseases are scarce [75], while several associations were observed with other diseases, especially cancer [76,77]. On the other hand, catalase activity was consistently decreased in erythrocytes of ALS patients [78,79,80], suggesting its important role in oxidative stress response; however, further studies are needed to validate the role of catalase in ALS.

We were the first to comprehensively evaluate the role of common polymorphisms in antioxidant and inflammation genes in ALS susceptibility and pathogenesis. Our study group reflects well the characteristics of SALS patients. *C9orf72* mutation was found in 3.2% of patients, similar to other studies [81]. Investigated clinical parameters in our study revealed shorter survival in older patients and shorter survival of patients with bulbar onset compared to spinal ALS onset, consistent with previous studies [82]. However, our study also has some limitations, mainly that ALS-FRS-R data were not available in all patients. Additionally, as *C9orf72* mutations are rare in SALS, we could not evaluate the role of investigated polymorphisms in carriers of this mutation separately. Another limitation was that controls were younger than ALS patients as only individuals younger than 65 years can serve as blood donors and that only data on gender and age were available for the control group. However, the possible relevance of the diverse age of the ALS patients and controls for the clinical course of the disease was taken into account while performing the adjustment in logistic regression analysis.

Taken together, we have shown for the first time that some of the selected studied polymorphisms in genes involved in inflammation (*IL1B* rs1071676) and oxidative stress (*SOD2* rs4880, *CAT* rs1001179), could be disease modifiers in ALS and that common genetic variants can influence ALS onset, rate of progression and survival. These types of studies are important to identify specific patient populations that may be responsive to the immune or antioxidant system—based therapies and to motivate future large-scale genetic research on modifiers of ALS progression for potential new treatments for ALS.

## Figures and Tables

**Table 1:** Clinical characteristics of ALS patients for the whole cohort (N = 185) and for patients with available ALS-FRS-R data (N = 164).

Characteristic	Category/Unit	Whole Cohort	Patients with ALS-FRS-R Data
Gender	Male, N (%)	94 (50.8)	84 (51.2)
	Female, N (%)	91 (49.2)	81 (48.8)
Age at onset	Years, Median (25–75%)	63 (56–71) [2]	63 (57–71)
Age at the time of blood collection	Years, Median (25–75%)	65 (58–72) [2]	65 (59–73) [2]
ALS onset	Spinal	133 (72.3) [1]	121 (73.8)
	Bulbar	45 (24.5)	40 (24.4)
	Other	6 (3.3)	3 (1.8)
*C9orf72* mutation	No, N (%)	179 (96.8)	159 (97.0)
	Yes, N (%)	6 (3.2)	5 (3.0)
Death	No, N (%)	25 (14.0) [7]	24 (14.9) [3]
	Yes, N (%)	153 (86.0)	137 (85.1)
Survival	Months, Median (25–75%)	39.7 (26.6–68.4) [7]	42.3 (28.0–72.3) [3]
Follow-up time	Months, Median (25–75%)	104.9 (90.7–156.6) [7]	124.2 (89.7–156.6) [3]
Level of functional impairment ^a^	Points, Median (25–75%)	34.94 (29.50–39.99) [21]	34.94 (29.50–39.99)
Rate of disease progression ^b^	Median (25–75%)	−0.80 (−1.40 to −0.30) [37]	−0.80 (−1.40 to −0.30) [16]

^a^ ALS-FRS-R, ^b^ Slope of the linear regression line for ALS-FRS-R points per month. Number of missing data is presented in [] brackets. ALS, amyotrophic lateral sclerosis; ALS-FRS-R, ALS functional rating scale revised.

**Table 2:** Association between the investigated polymorphisms and ALS risk.

Gene	SNP	Genotype	OR (95% CI)	P	OR (95% CI)_adj_	P_adj_
*SOD2*	rs4880	CC	Reference		Reference	
		CT	1.07 (0.69–1.66)	0.753	1.01 (0.58–1.74)	0.978
		TT	1.06 (0.64–1.77)	0.810	0.84 (0.44–1.58)	0.586
		CT + TT	1.07 (0.71–1.62)	0.748	0.95 (0.57–1.58)	0.834
*CAT*	rs1001179	CC	Reference		Reference	
		CT	0.92 (0.63–1.35)	0.679	0.94 (0.57–1.53)	0.792
		TT	0.84 (0.38–1.87)	0.676	1.03 (0.38–2.74)	0.958
		CT + TT	0.91 (0.63–1.32)	0.618	0.95 (0.60–1.51)	0.826
*GPX1*	rs1050450	CC	Reference		Reference	
		CT	0.95 (0.64–1.40)	0.788	1.05 (0.65–1.70)	0.849
		TT	0.97 (0.54–1.76)	0.921	1.11 (0.51–2.40)	0.788
		CT + TT	0.95 (0.66–1.37)	0.795	1.06 (0.67–1.67)	0.799
*IL1B*	rs1143623	GG	Reference		Reference	
		GC	0.80 (0.55–1.19)	0.272	0.74 (0.46–1.21)	0.235
		CC	1.07 (0.57–2.01)	0.832	0.93 (0.42–2.04)	0.850
		GC + CC	0.85 (0.59–1.23)	0.390	0.78 (0.49–1.23)	0.282
	rs16944	TT	Reference		Reference	
		TC	0.99 (0.56–1.76)	0.979	0.99 (0.48–2.04)	0.984
		CC	1.05 (0.59–1.85)	0.873	1.06 (0.52–2.17)	0.872
		TC + CC	1.02 (0.60–1.74)	0.942	1.03 (0.52–2.01)	0.939
	rs1071676	GG	Reference		Reference	
		GC	0.93 (0.63–1.37)	0.714	1.11 (0.68–1.80)	0.685
		CC	0.47 (0.19–1.21)	0.118	0.54 (0.18–1.61)	0.269
		GC + CC	0.86 (0.59–1.25)	0.424	1.01 (0.63–1.60)	0.981
*MIR146A*	rs2910164	GG	Reference		Reference	
		GC	1.05 (0.72–1.55)	0.793	0.85 (0.52–1.39)	0.518
		CC	0.74 (0.32–1.75)	0.498	0.54 (0.18–1.62)	0.273
		GC + CC	1.01 (0.70–1.46)	0.970	0.81 (0.50–1.29)	0.364
*IL6*	rs1800795	GG	Reference		Reference	
		GC	1.18 (0.78–1.77)	0.431	0.83 (0.49–1.39)	0.467
		CC	0.97 (0.58–1.63)	0.914	1.01 (0.53–1.91)	0.985
		GC + CC	1.11 (0.76–1.63)	0.584	0.88 (0.54–1.42)	0.593
*TNF*	rs1800629	GG	Reference		Reference	
		GA	0.88 (0.59–1.32)	0.542	0.97 (0.58–1.60)	0.897
		AA	0.94 (0.31–2.87)	0.916	0.77 (0.20–2.99)	0.707
		GA + AA	0.89 (0.60–1.31)	0.548	0.95 (0.58–1.54)	0.826

Adj: adjusted for age and gender; CI, confidence interval; OR, odds ratio; SNP, single nucleotide polymorphism.

**Table 3:** Comparison of genotype frequencies among patients with different types of ALS onset.

Gene	SNP	Genotype	Spinal N (%)	Bulbar N (%)	Other N (%)	*p*
*SOD2*	rs4880	CC	37 (80.4)	6 (13)	3 (6.5)	0.153
		CT	62 (68.1)	27 (29.7)	2 (2.2)	
		TT	33 (71.7)	12 (26.1)	1 (2.2)	
		CT + TT	95 (69.3)	39 (28.5)	3 (2.2)	Pdom = **0.036**
*CAT*	rs1001179	CC	81 (74.3)	23 (21.1)	5 (4.6)	0.585
		CT	44 (68.8)	19 (29.7)	1 (1.6)	
		TT	7 (70)	3 (30)	0 (0)	
		CT + TT	51 (68.9)	22 (29.7)	1 (1.4)	Pdom = 0.244
*GPX1*	rs1050450	CC	71 (76.3)	19 (20.4)	3 (3.2)	0.492
		CT	46 (64.8)	22 (31)	3 (4.2)	
		TT	16 (80)	4 (20)	0 (0)	
		CT + TT	62 (68.1)	26 (28.6)	3 (3.3)	Pdom = 0.446
*IL1B*	rs1143623	GG	76 (76)	23 (23)	1 (1)	0.249
		GC	44 (68.8)	16 (25)	4 (6.3)	
		CC	12 (63.2)	6 (31.6)	1 (5.3)	
		GC + CC	56 (67.5)	22 (26.5)	5 (6)	Pdom = 0.128
	rs16944	TT	15 (62.5)	6 (25)	3 (12.5)	0.063
		TC	55 (71.4)	19 (24.7)	3 (3.9)	
		CC	62 (75.6)	20 (24.4)	0 (0)	
		TC + CC	117 (73.6)	39 (24.5)	3 (1.9)	Pdom = 0.051
	rs1071676	GG	87 (77)	21 (18.6)	5 (4.4)	0.132
		GC	41 (64.1)	22 (34.4)	1 (1.6)	
		CC	4 (66.7)	2 (33.3)	0 (0)	
		GC + CC	45 (64.3)	24 (34.3)	1 (1.4)	Pdom = **0.039**
*MIR146A*	rs2910164	GG	80 (72.1)	28 (25.2)	3 (2.7)	0.665
		GC	48 (73.8)	14 (21.5)	3 (4.6)	
		CC	4 (57.1)	3 (42.9)	0 (0)	
		GC + CC	52 (72.2)	17 (23.6)	3 (4.2)	Pdom = 0.872
*IL6*	rs1800795	GG	43 (69.4)	17 (27.4)	2 (3.2)	0.655
		GC	61 (70.1)	23 (26.4)	3 (3.4)	
		CC	28 (82.4)	5 (14.7)	1 (2.9)	
		GC + CC	89 (73.6)	28 (23.1)	4 (3.3)	Pdom = 0.861
*TNF*	rs1800629	GG	91 (71.7)	32 (25.2)	4 (3.1)	1.000
		GA	37 (72.5)	12 (23.5)	2 (3.9)	
		AA	4 (80)	1 (20)	0 (0)	
		GA + AA	41 (73.2)	13 (23.2)	2 (3.6)	Pdom = 0.951

SNP, single nucleotide polymorphism; Pdom: *p*-value for dominant genetic model. The bold represents statistical significance.

**Table 4:** Association of investigated SNPs with level of functional impairment and rate of disease progression.

Gene	SNP	Genotype	Level of Functional Impairment Median (25–75%)	*p*	Rate of Disease Progression Median (25–75%)	*p*
*SOD2*	rs4880	CC	36.94 (31.07–40.86)	0.199	−0.66 (−1.25 to −0.27)	0.455
		CT	34 (29–39.6)		−0.77 (−1.47 to −0.28)	
		TT	34.18 (28.55–39.53)		−0.97 (−1.70 to −0.40)	
		CT + TT	34 (29–39.6)	Pdom = 0.073	−0.83 (−1.50 to −0.31)	Pdom = 0.263
*CAT*	rs1001179	CC	35 (29.21–39.98)	0.111	−0.81 (−1.42 to −0.34)	0.584
		CT	33.89 (29.06–39.23)		−0.71 (−1.54 to −0.28)	
		TT	39.29 (34.7–43.84)		−0.42 (−1.56 to −0.11)	
		CT + TT	34.67 (30.1–40.31)	Pdom = 0.690	−0.67 (−1.49 to −0.27)	Pdom = 0.847
*GPX1*	rs1050450	CC	34.75 (30–40.71)	0.606	−0.66 (−1.31 to −0.33)	0.323
		CT	35 (28.62–39.17)		−0.83 (−1.50 to −0.25)	
		TT	34.51 (29.75–40.51)		−1.12 (−2.27 to −0.47)	
		CT + TT	35 (29.12–39.48)	Pdom = 0.470	−0.92 (−1.57 to −0.26)	Pdom = 0.421
*IL1B*	rs1143623	GG	34.02 (29.5–40)	0.887	−0.72 (−1.34 to −0.25)	0.498
		GC	35.14 (29–39.83)		−0.76 (−1.75 to −0.32)	
		CC	34.93 (29.66–40.82)		−0.93 (−1.54 to −0.49)	
		GC + CC	35.14 (29.38–39.83)	Pdom = 0.843	−0.80 (−1.56 to −0.33)	Pdom = 0.307
	rs16944	TT	33.13 (29.57–40.3)	0.903	−1.04 (−1.50 to −0.53)	0.383
		TC	34.88 (28.74–39.83)		−0.66 (−1.35 to −0.28)	
		CC	35 (30–40)		−0.82 (−1.38 to −0.21)	
		TC + CC	35 (29–40)	Pdom = 0.893	−0.71 (−1.36 to −0.27)	Pdom = 0.174
	rs1071676	GG	34.39 (29.86–40.06)	0.565	−0.71 (−1.24 to −0.34)	**0.015**
		GC	34.73 (28.99–39.99)		−0.80 (−1.48 to −0.18)	
		CC	36.96 (34.52–40.12)		−2.40 (−3.10 to −1.69)	
		GC + CC	35 (29–39.98)	Pdom = 0.875	−0.81 (−1.77 to −0.24)	Pdom = 0.690
*MIR146A*	rs2910164	GG	34.73 (29.86–39.78)	0.548	−0.68 (−1.54 to −0.27)	0.695
		GC	34.38 (28.88–40.01)		−0.83 (−1.45 to −0.41)	
		CC	37.8 (31.05–41)		−0.83 (−1.17 to −0.19)	
		GC + CC	35 (29.12–40.09)	Pdom = 0.851	−0.83 (−1.39 to −0.39)	Pdom = 0.479
*IL6*	rs1800795	GG	34 (30–39.6)	0.285	−0.53 (−1.33 to −0.18)	0.216
		GC	34 (28–39.83)		−0.83 (−1.49 to −0.37)	
		CC	36.95 (32.08–40.4)		−0.83 (−1.64 to −0.28)	
		GC + CC	34.94 (29–40.2)	Pdom = 0.634	−0.83 (−1.52 to −0.34)	Pdom = 0.108
*TNF*	rs1800629	GG	35.04 (30.53–40.24)	0.512	−0.82 (−1.39 to −0.31)	0.827
		GA	34 (27.95–37.98)		−0.67 (−2.07 to −0.27)	
		AA	38.15 (27.46–39.98)		−0.80 (−0.81 to −0.36)	
		GA + AA	34 (27.91–38.54)	Pdom = 0.272	−0.68 (−1.49 to −0.27)	Pdom = 0.962

SNP, single nucleotide polymorphism; Pdom: P-value for dominant genetic model. The bold represents statistical significance.

**Table 5:** Association of investigated SNPs with survival of ALS patients.

Gene	SNP	Genotype	Median Survival (25–75%)	HR (95% CI)	*p*	HR (95% CI)_adj_	P_adj_
*SOD2*	rs4880	CC	37.6 (25.7–76.0)	Reference		Reference	
		CT	39.7 (27.7–69.2)	1.00 (0.67–1.50)	0.985	1.16 (0.71–1.9)	0.558
		TT	43.5 (23.7–63.0)	1.20 (0.76–1.87)	0.435	1.18 (0.69–2.03)	0.549
		CT + TT	40.7 (26.9–66.9)	1.06 (0.73–1.55)	0.742	1.17 (0.73–1.86)	0.520
*CAT*	rs1001179	CC	37.0 (26.6–65.8)	Reference		Reference	
		CT	43.7 (26.5–72.3)	0.83 (0.58–1.17)	0.283	0.70 (0.46–1.04)	0.079
		TT	60.3 (56.7–85.6)	0.61 (0.30–1.26)	0.179	0.62 (0.28–1.36)	0.231
		CT + TT	47.6 (26.5–78.6)	0.79 (0.57–1.1)	0.156	0.68 (0.47–0.99)	**0.046**
*GPX1*	rs1050450	CC	39.0 (28.2–69.2)	Reference		Reference	
		CT	39.7 (25.3–72.8)	0.89 (0.63–1.26)	0.507	0.85 (0.58–1.24)	0.396
		TT	40.5 (29.0–65.8)	0.99 (0.58–1.68)	0.969	1.06 (0.53–2.14)	0.869
		CT + TT	39.7 (25.3–67.6)	0.91 (0.66–1.26)	0.570	0.88 (0.61–1.26)	0.477
*IL1B*	rs1143623	GG	41.8 (26.5–74.5)	Reference		Reference	
		GC	39.0 (25.3–68.4)	1.12 (0.8–1.58)	0.503	1.21 (0.82–1.78)	0.332
		CC	37.0 (34.0–56.7)	1.32 (0.76–2.31)	0.322	0.78 (0.38–1.59)	0.491
		GC + CC	39.0 (27.7–67.6)	1.16 (0.84–1.6)	0.361	1.11 (0.77–1.6)	0.572
	rs16944	TT	37.0 (33.2–56.7)	Reference		Reference	
		TC	43.7 (25.7–76.0)	0.66 (0.4–1.11)	0.118	1.08 (0.55–2.09)	0.827
		CC	39.4 (26.5–66.9)	0.74 (0.45–1.23)	0.245	1.12 (0.59–2.13)	0.722
		TC + CC	41.8 (26.5–72.3)	0.70 (0.44–1.14)	0.150	1.1 (0.59–2.06)	0.754
	rs1071676	GG	39.7 (25.7–66.9)	Reference		Reference	
		GC	41.8 (28.7–83.6)	0.75 (0.53–1.07)	0.110	1.03 (0.69–1.53)	0.900
		CC	24.3 (18.4–32.0)	1.53 (0.62–3.77)	0.358	5.02 (1.92–13.16)	**0.001**
		GC + CC	40.5 (28.0–78.6)	0.79 (0.57–1.11)	0.171	1.13 (0.77–1.66)	0.529
*MIR146A*	rs2910164	GG	42.9 (25.7–72.3)	Reference		Reference	
		GC	39.0 (27.7–65.8)	1.03 (0.73–1.45)	0.860	1 (0.68–1.46)	0.989
		CC	36.7 (31.4–60.3)	1.26 (0.55–2.88)	0.584	1.42 (0.57–3.54)	0.448
		GC + CC	39.0 (27.7–65.8)	1.05 (0.76–1.46)	0.768	1.03 (0.71–1.49)	0.876
*IL6*	rs1800795	GG	37.0 (26.6–78.6)	Reference		Reference	
		GC	39.7 (25.7–60.3)	1.02 (0.84–1.73)	0.317	1.06 (0.69–1.62)	0.793
		CC	40.7 (29.9–74.5)	0.90 (0.56–1.43)	0.647	0.84 (0.48–1.46)	0.527
		GC + CC	40.5 (26.5–66.9)	1.10 (0.78–1.55)	0.585	1 (0.66–1.5)	0.984
*TNF*	rs1800629	GG	39.0 (25.7–67.6)	Reference		Reference	
		GA	41.8 (29.8–74.5)	0.92 (0.64–1.33)	0.670	0.78 (0.5–1.21)	0.266
		AA	37.0 (32.5–57.6)	1.25 (0.51–3.06)	0.631	1.45 (0.58–3.58)	0.426
		GA + AA	40.7 (29.8–74.5)	0.95 (0.67–1.35)	0.778	0.84 (0.56–1.27)	0.411

Adj: adjusted for age at onset and rate of disease progression. The bold represents statistical significance.

## Data Availability

The datasets during and/or analyzed during the current study available from the corresponding author on reasonable request.

## Supplementary Materials

The following supporting information can be downloaded at: https://www.mdpi.com/article/10.3390/genes13050757/s1, Table S1: Genotype frequencies of selected polymorphisms.
