# Supplementary material for: Genetic Variability of Inflammation and Oxidative Stress Genes Affects Onset, Progression of the Disease and Survival of Patients with Amyotrophic Lateral Sclerosis
Source: Genes (Basel). 2022 Apr 25;13(5):757. doi: 10.3390/genes13050757 (PMC9140599; doi:10.3390/genes13050757)
Supplement: Supplementary file 1 [file genes-13-00757-s001.zip › genes-1659516-supplementary.pdf]

Supplementary table S1: Genotype frequencies of selected polymorphisms.

**Gene**	**SNP**	**Role**	**Genotype**	**Controls N (%)**	**ALS N (%)**	**MAF (controls)**	**pHWE (controls)**
SOD2	rs4880	p.Ala16Val	CC	87 (26.9)	47 (25.5) [1]	0.489	0.584
			CT	157 (48.5)	91 (49.5)		
			TT	80 (24.7)	46 (25.0)		
CAT	rs1001179	c.-262C>T	CC	184 (5.0) [1]	109 (59.2) [1]	0.246	0.897
			CT	119 (36.8)	65 (35.3)		
			TT	20 (6.2)	10 (5.4)		
GPX1	rs1050450	p.Pro198Leu	CC	159 (49.1)	93 (50.3)	0.312	0.153
			CT	128 (39.5)	71 (38.4)		
			TT	37 (11.4)	21 (11.4)		
IL1B	rs1143623	c.-1560G>C	GG	165 (50.9)	101 (54.9) [1]	0.290	0.641
			GC	130 (40.1)	64 (34.8)		
			CC	29 (9)	19 (10.3)		
	rs16944	c.-598T>C	TT	43 (13.3)	24 (13.0) [1]	0.653*	0.334
			TC	139 (42.9)	77 (41.8)		
			CC	142 (43.8)	83 (45.1)		
	rs1071676	c.*505G>C	GG	189 (58.3)	114 (62.0) [1]	0.219	0.305
			GC	114 (35.2)	64 (34.8)		
			CC	21 (6.5)	6 (3.3)		
MIR146A	rs2910164	n.60G>C	GG	196 (60.5)	111 (60.3) [1]	0.227	0.461
			GC	109 (33.6)	65 (35.3)		
			CC	19 (5.9)	8 (4.3)		
IL6	rs1800795	c.-174G>C	GG	117 (36.1)	62 (33.7) [1]	0.421	0.053
			GC	141 (43.5)	88 (47.8)		
			CC	66 (20.4)	34 (18.5)		
TNF	rs1800629	c.-308 G>A	GG	217 (67)	128 (69.6) [1]	0.179	0.601
			GA	98 (30.2)	51 (27.7)		
			AA	9 (2.8)	5 (2.7)		

Number of missing data is presented in [] brackets.
*polymorphic allele more common in European populations
